# Exploring the Vasculitis‐Tumors Link: Epidemiological Patterns, Mechanistic Insights, and Clinical Implications

**DOI:** 10.1002/advs.202521056

**Published:** 2026-02-03

**Authors:** Xiaofei Shi, Zhijiao Li, Xin Ma, Bei Zhang, Xinfeng Wu, Hua Fan, Rongzeng Liu, Hongwei Jiang, Cong‐Qiu Chu

**Affiliations:** ^1^ Department of Rheumatology and Immunology The First Affiliated Hospital, and College of Clinical Medicine of Henan University of Science and Technology Luoyang China; ^2^ Office of Research & Innovation The First Affiliated Hospital College of Clinical Medicine of Henan University of Science and Technology Luoyang China; ^3^ Department of Immunology College of Basic Medicine and Forensic Medicine Henan University of Science and Technology Luoyang China; ^4^ Henan Key Laboratory of Rare Diseases Endocrinology and Metabolism Center The First Affiliated Hospital, and College of Clinical Medicine of Henan University of Science and Technology Luoyang China; ^5^ Division of Arthritis and Rheumatic Diseases Oregon Health & Science University VA Portland Health Care System Portland Oregon USA

**Keywords:** causality, clinical diagnosis and treatment, pathogenesis, tumor, vasculitis

## Abstract

This review systematically examines the association between vasculitis and malignancies by summarizing the risk of tumor‐associated vasculitis, their clinical characteristics, and proposed pathogenic mechanisms across different vasculitic subtypes. Relevant case reports and mechanistic studies are integrated to provide a comprehensive overview of this entity. The findings offer practical value for rheumatologists in distinguishing primary vasculitis from malignancy‐associated forms and optimizing patient surveillance, and for oncologists in improving recognition of tumor‐associated vasculitis to reduce the risk of misdiagnosis, supporting more accurate clinical decision‐making and risk management.

## Graphical abstract

This review outlines association between vasculitis and malignancies and provides practical value for clinicians in distinguishing primary vasculitis from malignancy‐associated forms and optimizing patient surveillance, improving recognition of tumor‐associated vasculitis to reduce the risk of misdiagnosis, supporting more accurate clinical decision‐making and risk management.

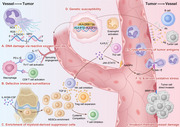

## 1 Introduction

Vasculitis comprises a heterogeneous spectrum of disorders defined by inflammatory injury to blood vessel walls, resulting in systemic or organ‐specific manifestations. Such vascular damage can compromise tissue perfusion and precipitate dysfunction in multiple organs, significantly affecting patients’ quality of life and long‐term prognosis. Malignancy remains the second leading cause of death worldwide, with approximately 20 million new cancer cases reported in 2022 and projections indicating a rise to 35 million cases by 2050 [1]. Research suggests that the coexistence of vasculitis and cancer is not coincidental. Clinical and population‐based studies have consistently reported a higher incidence of malignancy among patients with various vasculitic conditions than in the general population (Table 1). Moreover, an increasing number of case reports describe patients with cancer who subsequently develop bona fide vasculitic syndromes (Table 2), suggesting that tumors may serve as immunologic or inflammatory triggers.

**TABLE 1:** Main Characteristics of Studies Focused on Cancer Incidence After Vasculitis Diagnosis.

	Time period	Location	Study design	Vasculitis type	Study population	Cancer cases	SIR/HR (95% CI)
Han et al., 2024 [5]	1995‐2021	China	Single‐center retrospective cohort study	AAV^a^	529	43	2.24(95% CI: 1.68–2.99)
Lee et al., 2022 [17]	2009–2019	South Korea	Nationwide database retrospective study	TAK	1449	74	1.62(95%CI:1.27–2.03)
Stamatis et al., 2020 [9]	1997–2010	Sweden	Single‐center retrospective cohort study	GCA	830	107	0.98(95%CI:0.81–1.17)
Guven et al., 2020 [13]	2014–2020	Turkey	Single‐center retrospective study	BD	451	11	2.84(95%CI:1.50–4.94)
Wang et al., 2015 [16]	2000–2009	China	Taiwan Provincial database retrospective study	BD	1314	30	1.50(95%CI:1.03–2.11)

^a^ AAV: Antineutrophil cytoplasmic antibody‐associated vaculitis; BD: Behcet's disease; GCA: Giant cell arteritis; HR: Hazard ratio; SIR: Standardized incidence ratio; TAK: Takayasu arteritis.

**TABLE 2:** Summary of Clinical Features of Tumor‐Induced Vasculitis.

Tumor type		Vasculitis Type	Demographics		Clinical manifestation		Laboratory/imaging data		Treatment	Outcome /Prognosis
			Age Sex	Country	Systemic	Cutaneous	Laboratory	Imaging		
Hematological neoplasms										
Leukemia	Broccoli et al., 2016 [58]	LCV^a^	37 M	Italy	Fever, splenomegaly, pancytopenia	Leg purpura, confluent scalp and trunk plaques	Pancytopenia; skin biopsy: LCV	/	Prednisone/ Cladribine (LCV cleared at week 3)/Rituximab	LCV‐CR; hematologic CR only after rituximab
	Atallah et al., 2017 [60]	LCV	74 M	USA	/	12cm ulcerated ankle tumor	Leukocytosis; Neutrophilia; bone marrow: CML	/	Dapsone/ Hydroxyurea/Prednisone	Tumor regressed; died of sepsis (chemo‐induced neutropenia)
	Sasinowska et al., 2017 [74]	Aortitis	68 F	USA	Dyspnea, fatigue, fever, night sweats, weight loss	Left breast and trunk pink dermal papulonodules	ANA:1:640; ESR/CRP↑; Leukocytosis; bone marrow: CMML	PET‐CT: Thoracoabdo‐minal aortic thickening	Prednisone/ Azacitidine.	Died due to leukemia progression
	Moyers et al., 2019 [59]	LCV	62 F	USA	Fatigue, fever, bruising, epistaxis	Generalized purpuric papules	Bone marrow: HCL; skin biopsy: LCV	/	Cladribine	Rash and leukemia CR, sustained remission at 14 months
	Çetinarslan et al., 2020 [75]	LCV	57 M	Turkey	/	Annular erythematous plaques on arms	Pancytopenia; bone marrow: AML‐M4.	/	Cytarabine/ potent topical steroids	Skin CR, died of sepsis 8 months later
	Kolodziejc‐zyk et al., 2021 [61]	cPAN	78 F	USA	/	Generalized purpuric papules	Leukocytosis; thrombocytosis; c‐ANCA(+)	CT: no systemic vasculitis	Imatinib	Complete resolution of lesions, no recurrence in 27 months
HL	Lopez‐Chiriboga et al., 2018 [62]	Granulomatous Angiitis of CNS	25 F	USA	Headache.	/	CSF protein↑, lymphocytic pleo‐cytosis; bone marrow: nodular sclerosing HL	MRI: White matter hyperintensiti‐es; PET: lymphadenop‐athy	Cyclophosph‐amide/ABVD	Neurological function and imaging improved
	Li et al., 2023 [63]	PACNS	44 M	Australia	Headache, cognitive decline, hyper‐ somnolence, fever, weight loss	/	CSF protein↑; lymphocytic pleocytosis; bone marrow: NLPHL	MRI: whitematter hyperintensiti‐es; PET: lymphadenop‐athy	ReCHOP	Cognitive improvement, MRI lesions reduced
MM	Oka et al., 2018 [71]	LCV	85 F	Japan	/	Vascular purpura on the lower limbs, chest, and abdomen	Leukocytosis (eosinophils 13.5×10^9^/L); anemia; IgG/κ↑; bone marrow: plasma cells 16.2%	/	VRD	no recurrence
Solid tumors										
Lung tumor	Aguiar et al., 2015 [76]	GCA	75 F	Brazil	Headache, neck/shoulder pain, SIADH	Thickened, tender right temporal artery	ESR↑; CRP↑	CT: 8 mm lung nodule	Thoracoscop‐ic resection, stop prednisone	Symptom free at 2 years, vasculitis resolved
	Pantoja Zarza et al., 2015 [77]	LCV	57 F	Spain	Arthralgia, abdominal pain, vomiting, weight loss	Palpable purpura on legs, acrocyanosis	ESR↑; CRP↑; Skin biopsy: LCV; CYFRA 21.1↑	CT: hilar lymphadenop‐athy	Prednisone/ cisplatin/ docetaxel	Tumor unresectable, vasculitis resolved in 10 days
	Bachmeyer et al., 2019 [66]	LCV	71 M	France	/	Ecchymoses on face/arms, palpable purpura on legs	Vitamin C↓; ANA:1:1280	PET/CT: hypermetabol‐ic nodule in left lower lobe	Vitamin C/ carboplatin/ p‐aclitaxel	Vasculitis and tumors improved
	Scopelliti et al., 2020 [65]	GCA	77 M	Italy	Dizziness, Vomiting, gait disturbance	/	ESR↑; CRP normal; brain DSA: multiple stenoses in the left vertebral artery	CT: left upper lobe tumor invading the right main bronchus	Methylprednisolone pulse/oral prednisone/ left upper lobectomy	Died of tumor progression with bone metastases 7 months post‐operation
	Taoka et al., 2021 [64]	IgAV	72 M	Japan	Fever, renal dysfunction	Palpable purpura on legs	Proteinuria: 476 mg/mmol; hematuria; renal biopsy: IgA mesangial deposition.	/	Methylpredn‐isolone pulse/ prednisone maintenance	Vasculitis resolved in 2 weeks, and the patient died of tumor progression
Esophag‐eal tumor	Ganchinho Lucas et al., 2021 [67]	Aortitis	80 M	Portugal	Fever, malaise, anorexia, migratory arthritis, dysphagia	/	WBC:9.7K/µL (neutrophils 82%); CRP↑; ESR↑	CT: aortic calcification, para‐aortic nodes; TEE: 7×1.5mm vegetation‐like mass; MRI: aortic wall thickening	Antibiotics (no effect)/ prednisolone (rapid relief)/ chemoradiat‐ion (unresectable)	Died months after cancer diagnosis
Renal cell tumor	Larbi et al., 2016 [78]	LCV	51 F	Tunisia	/	Recurrent purpuric lesions on lower limbs, necrotic purpura on ankles	CBC/HIV/ANA all normal	Uroscanner: 3 cm hyper‐vascularized mass in the right renal lower pole	Right renal tumorectomy	Complete resolution
	Massucci et al., 2021 [79]	LCV	66 F	Italy	/	/	ANCA negative	Brain MRI: 2‐cm hypodense lesion in the left rolandic area; Total body CT: adrenal metastases	Discontinued sunitinib/ Steroid therapy	Reduction of brain lesion, stability of metastatic sites
Prostate tumor	Figueiredo et al., 2021 [68]	GCA	73 M	Portugal	Night sweats, headache, thigh pain, low fever, mandibular claudication	/	Normocytic normochromic anemia; CRP↑; ESR↑	Temporal artery Doppler ultrasound: edematous halo	Prednisolone	Rapid relief; bone metastasis at 3 months
	Barbu et al., 2024 [80]	LCV	60 M	Romania	Dysuria, nycturia	Arm/hip necrotic ulcers	PSA↑	Bone scan: Bone metastases	Radiotherapy 76 Gy/ goserelin/ methylprednisolone	Lesions healed with residual pigment
Breast tumor	Dhana et al., 2018 [81]	LCV	53 F	South Africa	Painful lesions on limbs and trunk	Palpable purpura with necrosis/bullae/ulcers	Hb↓	Bone scan: T1 and R9 rib uptake	Dapsone/ Doxorubicin/ Cyclophosph‐amide/ Paclitaxel/ Radiotherapy	Skin healed; 6 months later cerebellar metastasis, craniotomy and palliative radiotherapy
	Esperança‐Martins et al., 2021 [82]	AAV	77 F	Portugal	Dry cough, fatigue, hemoptysis, breast mass, limb purpura 6 months ago	/	MPO‐ANCA↑; PR3‐ANCA↑; breast biopsy: papillary ductal carcinoma	CT: diffuse alveolar hemorrhage; Mammograp‐hy: right breast mass	Prednisone/ plasmaphere‐sis/Radical mastectomy	Death
	Mohamm‐ed et al., 2024 [69]	AAV	66 F	USA	Polyarticular pain, paresthesia, numbness, renal dysfunction	Petechial rash on lower limbs	P‐ANCA(+), MPO(+), breast biopsy: infiltrating ductal carcinoma, renal biopsy: crescentic pauci‐immune glomerulonephritis	/	Steroids/Rituximab/mastectomy	Symptoms resolved

^a^ ABVD: Adriamycin, Bleomycin, Vinblastine, Dacarbazine; AAV: Antineutrophil cytoplasmic antibody‐associated vaculitis; AML: Acute myeloid leukemia; ANA: Antinuclear antibody; CBC: Complete blood count; CML: Chronic myeloid leukemia; C: Complement; CR: Complete response; CRP: C‐reactive protein; CSF: Cerebrospinal fluid; CT: Computed tomography; cPAN: Cutaneous polyarteritis nodosa; CYFRA 21. 1: Cytokeratin fragment antigen 21‐1; CNS: Central nervous system; CCP: Cyclic citrullinated peptide; DSA: Digital subtraction angiography; dsDNA: Double‐stranded deoxyribonucleic acid; ESR: Erythrocyte sedimentation rate; F: Female; GPA: Granulomatosis with polyangiitis; GCA: Giant cell arteritis; HIV: Human immunodeficiency virus; HL: Hodgkin lymphoma; Ig: Immunoglobulin; IgAV: Immunoglobulin A vasculitis; LCV: Leukocytoclastic vasculitis; M: Male; MRI: Magnetic resonance imaging; MPO: Myeloperoxidase; NLPHL: Nodular lymphocyte‐predominant Hodgkin lymphoma; PACNS: Primary angiitis of the central nervous system; PET: Positron emission tomography; PR3: Proteinase 3; PSA: Prostate‐specific antigen; ReCHOP: Rituximab, Cyclophosphamide, Doxorubicin, Vincristine, Prednisone; RNP: Ribonucleoprotein; SIADH: Syndrome of inappropriate antidiuretic hormone secretion; TEE: Transesophageal echocardiography; VGPR: Very good partial response; VRD: Bortezomib, Lenalidomide, Dexamethasone.

Clarifying the relationship between vasculitis and malignancy is essential for improving diagnostic precision, optimizing therapeutic decision‐making, and ultimately enhancing patient outcomes in both rheumatology and oncology. However, the pathogenic mechanisms underlying this association remain incompletely understood, and standardized clinical management recommendations are still lacking. In this review, current epidemiological data are integrated and mechanistic hypotheses linking vasculitis with cancer, drawing on evidence from molecular and clinical investigations. The review further discusses the implications for clinical practice and future research directions, aiming to provide a conceptual framework to support precise diagnosis and targeted treatment in patients affected by this complex comorbidity.

## 2 Clinical Features and Pathophysiological Mechanisms of Tumor‐Associated Vasculitis

### 2.1 Tumor Risk Profiles in Different Vasculitis Subtypes

According to the 2012 revised Chapel Hill Consensus Conference nomenclature [2], vasculitic syndromes are classified into seven principal categories based on the predominant vessel size involved and underlying pathological characteristics: (1) large‐vessel vasculitis, such as Takayasu arteritis (TAK) and giant cell arteritis (GCA); (2) medium‐vessel vasculitis, exemplified by polyarteritis nodosa; (3) small‐vessel vasculitis, including antineutrophil cytoplasmic antibody–associated vasculitis (AAV); (4) variable‐vessel vasculitis, such as Behçet's disease (BD); (5) single‐organ vasculitis; (6) vasculitis associated with systemic diseases; and (7) vasculitis with a probable underlying cause. The risk of malignancy differs substantially among these categories, with the most consistent and robust epidemiological evidence reported for AAV, GCA, and BD. The following sections summarize the key epidemiological findings for each vasculitis group.

#### 2.1.1 Tumor Risk in AAV

AAV comprises a group of small‐vessel vasculitides, including granulomatosis with polyangiitis (GPA), microscopic polyangiitis (MPA), and eosinophilic granulomatosis with polyangiitis (EGPA). Numerous international cohort studies and meta‐analyses have demonstrated that patients with AAV carry a significantly higher risk of developing secondary malignancies than the general population, with clear heterogeneity across disease subtypes.

Large‐scale studies from different regions have uniformly reported an increased cancer risk in AAV. A meta‐analysis incorporating six European cohort studies, reported an overall standardized incidence ratio (SIR) for malignancy of 1.74 (95% CI 1.37–2.21) [3]. Similarly, a nationwide cohort study conducted in Korea identified an elevated cancer risk, with a hazard ratio (HR) of 1.32 (95% CI 1.08–1.61) [4]. A single‐center retrospective cohort study in China has also reported similar results, demonstrating that even after excluding individuals with a previous history of malignancy, the SIR for newly diagnosed tumors during follow‐up remained high at 2.24 (95% CI 1.68–2.99) [5]. These data confirm that the incidence of malignancy among patients with AAV is substantially higher than in the general population. Further support is provided by a multicenter meta‐analysis which pooled data from Europe, Asia, and Oceania and reported an overall cancer SIR of 1.767 (95% CI 1.514–2.063, *p* < 0.001). The highest risk was observed in Asian populations (SIR = 2.193), and the increased cancer risk persisted across age groups, with male patients demonstrating a significantly higher risk than female patients [6]. These observations have important implications for the development of risk‐adapted cancer screening strategies in clinical practice.

The spectrum of malignancy risk differs substantially among AAV subtypes. In GPA, multiple studies have demonstrated increased risks of non‐melanoma skin cancer, bladder cancer, and myeloid leukemia. A long‐term Danish cohort study of 293 individuals with GPA further showed that the SIR for non‐melanoma skin cancer reached 7.0 (95% CI 2.3–16) 20 years after diagnosis, while the SIRs for bladder cancer and myeloid leukemia peaked at 14.4 (95% CI 5.3–31) during 10–14 years and 23.9 (95% CI 2.7–86) during 5–9 years of follow‐up, respectively [7]. These findings were corroborated in a Korean cohort of 684 GPA patients which reported an HR of 7.39 for hematologic malignancies, including myeloid leukemia, and 4.20 for bladder cancer. In the same study, a significantly increased risk of lung cancer was observed in patients with MPA (HR = 2.86, 95% CI 1.25–6.55), whereas patients with EGPA displayed a significantly elevated risk of hematologic malignancies (HR = 4.65, 95% CI 2.03–10.67) [4]. However, in contrast to the robust evidence supporting malignancy risk in GPA, the subtype‐specific associations for MPA and EGPA have thus far been primarily derived from a single national cohort, highlighting the need for further independent studies to define tumor risks across these AAV subtypes more comprehensively.

Through a systematic review of recent cohort studies, Wester Trejo et al. delineated the differential effects of immunosuppressive therapies on malignancy risk in AAV. Cyclophosphamide was shown to exert an apparent dose‐dependent carcinogenic effect, with cumulative exposures exceeding 36 g being associated with a significantly increased cancer risk. Rituximab appeared to confer a protective effect, as the incidence of malignancy in rituximab‐treated patients was comparable to that of the general population (standardized incidence ratio = 0.67). Higher cumulative doses of rituximab (>6.0 g) were associated with a potential protective effect, corresponding to a 6.32‐fold reduction in cancer risk. As the clinical use of cyclophosphamide has declined, the risk of multi‐organ carcinogenesis has decreased substantially; however, with the widespread adoption of azathioprine for maintenance therapy, non‐melanoma skin cancer has emerged as the predominant malignancy risk in current practice [8].

#### 2.1.2 Tumor Risk in GCA

GCA is a granulomatous form of large‐vessel vasculitis that primarily involves the aorta and its major branches. Although studies have yielded variable conclusions regarding the overall risk of malignancy in patients with giant cell arteritis, there is a broad consensus that the incidence of hematologic malignancies, particularly myeloid leukemia, is increased in this population.

In a population‐based study from southern Sweden, Stamatis et al. evaluated 830 biopsy‐confirmed cases of GCA and found no significant elevation in overall cancer risk (SIR = 0.98); however, the risk of myeloid leukemia was significantly increased (SIR = 2.31) [9]. Similarly, a study conducted in the Côte‐d'Or region of France reported that male patients with GCA showed significantly higher risks of myeloid hematologic malignancies (SIR = 4.82) and myeloproliferative neoplasms (SIR = 9.04) among 276 biopsy‐proven cases [10]. Ješe et al. in Slovenia conducted a prospective cohort study enrolling 107 patients with GCA. The results showed that the overall risk of malignant neoplasms in GCA patients was significantly elevated (SIR = 4.61). Bladder cancer was found to be the predominant malignancy type, and smokers had a higher risk of developing bladder cancer. However, the reliability of the study's conclusions is limited by its relatively small sample size [11].

Despite methodological differences and potential regional variability that may account for discrepancies in overall cancer risk estimates, a consistent pattern emerges across studies linking GCA with malignancies of the myeloid hematopoietic system. These observations underscore the importance of vigilant hematologic surveillance in patients with GCA, particularly among those with established risk factors such as advanced age or a history of smoking.

#### 2.1.3 Tumor Risk in BD

BD, categorized as a variable‐vessel vasculitis, is characterized by inflammatory involvement of both arterial and venous vessels. It manifests with recurrent oral and genital ulcers and may involve multiple organ systems, including the eyes, skin, joints, gastrointestinal tract, and central nervous system. Referred to as the “Silk Road disease” because of its high prevalence in the Mediterranean basin, the Middle East, and East Asia [12], epidemiological evidence indicates that patients with BD has a significantly higher risk of malignancy than the general population, with significant geographic variation and time‐dependent patterns.

Most available data originate from regions with a high disease prevalence. A retrospective cohort study from Turkey including 451 patients, reported a significantly elevated SIR for malignancy of 2.84 (95% CI 1.50–4.94) in patients with BD. Cancer risk was substantially higher in male patients (SIR = 5.63, 95% CI 2.62–10.70, *p* < 0.001), whereas no significant increase was observed among females (SIR = 1.22, 95% CI 0.31–3.33, *p* = 0.68). Owing to the limited number of cancer cases, the study was unable to delineate associations with specific tumor types but suggested that azathioprine therapy might reduce malignancy risk (p = 0.031), implying a potential protective effect through inflammation control [13].

Evidence from East Asian populations has further highlighted the dynamic nature of cancer risk in BD. A nationwide Korean cohort with a median follow‐up of 2.34 yearsreported significantly increased overall cancer risks, with an SIR of 3.54 (95% CI 2.35–5.11) in males and 2.17 (95% CI 1.58–2.92) in females. Among male patients, colorectal (SIR = 4.26) and liver cancers (SIR = 4.00) were most prevalent. Female patients showed significantly increased risks of oropharyngeal (SIR = 13.97) and liver malignancies (SIR = 12.78). Myelodysplastic syndrome showed a strikingly high incidence, with an SIR of 65.72 in males and 53.86 in females [14]. In comparison, longer‐term follow‐up data (≥5 years) from another Korean population‐based study demonstrated a persistent elevation in cancer risk over time, with an overall HR of 1.134 and particularly pronounced risks for hematologic malignancies, including leukemia (HR = 5.801) and lymphoma (HR = 2.584) [15]. Consistent with these findings, a cohort study in Taiwan with a mean follow‐up of seven years, reported increased SIR for non‐Hodgkin lymphoma (8.33) and breast cancer (2.16) in female patients with Behçet's disease, while no statistically significant increase in malignancy risk was observed among male patients [16].

These studies suggest a temporal evolution in malignancy risk among patients with BD, characterized by a predominance of solid tumors in the early disease course and a subsequent increase in hematologic malignancies over time. This shifting risk profile highlights the importance of implementing time‐adapted cancer surveillance strategies in this population. Further multicenter and cross‐regional investigations are warranted to clarify the contributions of ethnic, genetic, and therapeutic factors to cancer susceptibility in BD.

#### 2.1.4 Cancer Risk in Other Forms of Vasculitis

Although less extensively investigated, several other vasculitic disorders have also been associated with an increased risk of malignancy. TAK and polyarteritis nodosa, for example, have been linked to specific tumor types, primarily based on small case series and individual reports. In a Korean study, Lee et al. described rare cases of TAK complicated by secondary myelodysplastic syndrome, breast cancer, and ovarian cancer, suggesting a potential predisposition to malignancy in this patient population [17]. Similarly, a French study reported a higher incidence of hematologic malignancies among patients with polyarteritis nodosa (PAN), further supporting a possible association between chronic vascular inflammation and hematopoietic neoplasia [18]. Although derived from limited cohorts, these observations provide meaningful insights and reinforce the notion that distinct vasculitic syndromes may contribute to tumorigenesis via distinct pathogenic pathways. Further studies are needed to define the cancer risks specific to each vasculitis subtype.

Malignancy risk profiles vary substantially across vasculitic conditions, with well‐characterized patterns observed in major entities such as AAV, GCA, and BD, and more limited but suggestive evidence available for TAK and PAN. The incidence rates of concurrent malignancies and corresponding SIR across different vasculitis types are summarized in Table 3, allowing direct comparison of cancer risk levels among disease subgroups and providing quantitative support for the conclusions discussed above.

**TABLE 3:** Number of Cancers and Corresponding Standardized Incidence Ratios in Vasculitis Studies.

Cancer Site	Han et al., 2024 [5]		Lee et al., 2022 [17]		Na et al., 2018 [15]	
	N^a^	SIR (95% CI)	N	SIR (95% CI)	N	HR (95% CI)
All Cancers	43	2.24(1.68‐2.99)	74	1.62 (1.27–2.03)	451	1.134 (1.029‐1.25)
Digestive System						
Tongue	0	/	0	/	0	/
Esophagus	0	/	0	/	1	0.534 (0.071‐3.993)
Gastric	2	0.89 (0.22–3.55)	4	0.82 (0.22–2.09)	31	0.648 (0.451‐0.932)
Pancreas	0	/	0	/	21	1.192 (0.757‐1.877)
Liver/Bile Ducts	2	1.16 (0.29–4.65)	0	/	35	/
Colorectal	1	1.03 (0.14–7.38)	0	/	56	0.859 (0.653‐1.129)
Respiratory System						
Lip/Oral Cavity/Pharynx	0	/	0	/	11	2.113 (1.102‐4.052)
Lung	22	5.01 (3.29–7.62)	8	2.18 (0.94–4.29)	33	1.065 (0.743‐1.528)
Central Nervous System						
Brain/Spinal cord	0	/	0	/	14	1.735 (0.982‐3.064)
Urinary System						
Kidney	2	8.00 (1.90–33.69)	3	3.96 (0.82–11.58)	14	1.628 (0.922‐2.873)
Bladder	2	4.76 (1.16–19.62)	0	/	11	1.773 (0.931‐3.374)
Ureter	2	45.46 (11.05–187.02)	0	/	0	/
Renal Pelvis	1	27.78 (3.82–202.23)	0	/	0	/
Reproductive System						
Prostate	2	4.65(1.13–19.15)	0	/	23	1.784 (1.141‐2.791)
Testis	1	175.44(23.39–1264.63)	0	/	0	/
Ovary	1	4.35 (0.59–32.17)	4	4.45 (1.21–11.39)	14	1.157 (0.665‐2.013)
Uterus	0	/	4	1.63 (0.20–5.89)	0	/
Cervix	1	2.38 (0.33–17.27)	2	2.07 (1.16–3.42)	9	0.586 (0.299‐1.148)
Breast	1	0.85 (0.12–6.06)	15	2.07 (1.16–3.42)	57	0.89 (0.679‐1.167)
Hematological/ Immunological System						
Thymus	1	19.23 (2.66–138.85)	0	/	0	/
Lymphoma	1	3.33 (0.35–31.99)	2	/	19	2.584 (1.559‐4.283)
Leukemia	0	/	2	5.45(0.66–19.69)	18	5.801 (3.24‐10.385)
MM	0	/	1	3.56(0.09–19.82)	4	1.352 (0.474‐3.852)
MDS	0	/	3	18.02(3.712–52.66)	0	/
Endocrine System						
Thyroid	0	/	14	1.44 (0.79–2.42)	136	1.256 (1.05‐1.501)
Skin						
Non‐melanoma skin cancer	1	6.25 (0.83–47.04)	0	/	2	0.863 (0.203‐3.676)
Melanoma	0	/	0	/	0	/

^a^ HR: Hazard ratio; MDS: Myelodysplastic syndromes; MM: Multiple myeloma; N: Number; SIR: Standardized incidence ratio.

### 2.2 Potential Mechanisms of Cancer Development Associated with Vasculitis

**FIGURE 1:**
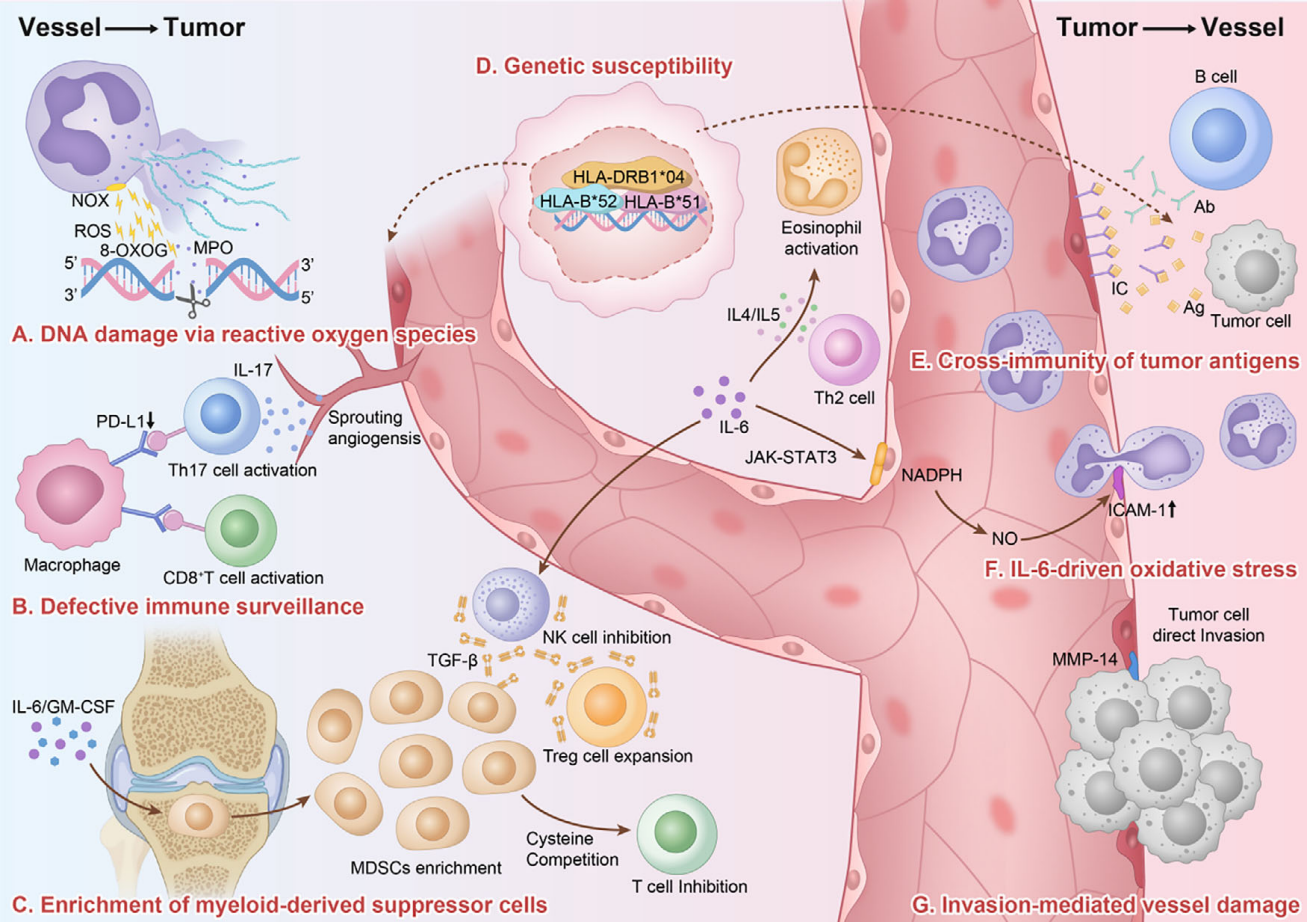
Mechanisms of tumor development in vasculitis and vasculitis induction by tumors. Ab: Antibody; Ag: Antigen; GM‐CSF: Granulocyte‐macrophage colony‐stimulating factor; HLA: Human leukocyte antigen; IC: Immune complex; ICAM: Intercellular adhesion molecule; IL: Interleukin; JAK: Janus kinase; MDSCs: Myeloid‐derived suppressor cells; MMP: Matrix metalloproteinase; MPO: Myeloperoxidase; NADPH: Nicotinamide adenine dinucleotide phosphate; NOX: NADPH oxidase; NO: Nitric oxide; NK: Natural killer cell; 8‐oxoG: 8‐Oxoguanine; PD‐L1: Programmed death‐ligand 1; ROS: Reactive oxygen species; STAT: Signal transducer and activator of transcription; TGF‐β: Transforming growth factor‐β; Th17: T helper 17 cell; Treg: Regulatory T cell. (This figure was created using Adobe Illustrator 2020, without the use of AI‐generated design tools).

The recurrent coexistence of vasculitis and malignancy indicates that their association is unlikely to be coincidental. Increasing mechanistic evidence points to three closely interconnected processes underlying this relationship: chronic inflammation–driven oxidative damage, immune dysregulation accompanied by compromised tumor immune surveillance, and shared genetic predispositions. These pathways constitute a coherent biological framework through which vasculitis may increase susceptibility to malignant transformation (Figure 1).

#### 2.2.1 Chronic Inflammation and Oxidative Stress

Persistent inflammation is a well‐recognized driver of carcinogenesis, mainly through the cumulative accrual of DNA damage mediated by oxidative stress. Inflammatory activation of neutrophils leads to the generation of reactive oxygen species (ROS) via nicotinamide adenine dinucleotide phosphate (NADPH) oxidase (NOX). These ROS induce oxidative DNA lesions, such as 8‐oxoguanine, and promote DNA strand breaks, facilitating mutagenesis [19, 20]. Neutrophil extracellular traps (NETs), which consist of myeloperoxidase (MPO)–elastase complexes, further exacerbate chromatin damage within inflamed tissues [21]. This inflammatory–oncogenic cascade has been well documented in chronic inflammatory disorders, including hepatitis and inflammatory bowel disease, both of which are associated with an increased risk of malignancy [22, 23].

More than 80%–85% of patients with AAV show pulmonary nodules and other lung lesions considered to confer a high risk for lung cancer [24]. In this setting, NET formation is significantly enhanced in the lungs, and MPO, which serves both as a major structural component of NETs and as a key autoantigen targeted by antineutrophil cytoplasmic antibodies, contributes to the pathogenesis of interstitial lung disease [25]. Supporting its carcinogenic potential, studies in smoking populations have demonstrated a significant positive correlation between pulmonary MPO activity and DNA adduct formation (*p* = 0.003) [26], indicating that MPO‐driven oxidative injury may play a direct role in inflammation‐associated tumorigenesis (Figure 1).

#### 2.2.2 Impaired Immune Surveillance

During the progression of vasculitis, disruptions in immune surveillance facilitate tumor immune escape by altering the balance between immune activation and suppression. In antineutrophil cytoplasmic antibody–associated vasculitis and giant cell arteritis, the chronic inflammatory milieu is related to dysfunction of the programmed death‐1/programmed death‐ligand 1 (PD‐1/PD‐L1) axis, leading to excessive activation and impaired regulation of effector T‐cell populations, including CD8^+^ T cells and T helper 17 (Th17) cells [27, 28, 29]. Activated Th17 cells secrete interleukin (IL)‐17, which promotes angiogenesis and extracellular matrix remodeling, increasing tumor invasiveness [30, 31, 32]. Similarly, inflammatory mediators such as IL‐6 and granulocyte‐macrophage colony‐stimulating factor (GM‐CSF) drive the differentiation and tissue infiltration of myeloid‐derived suppressor cells. These cells inhibit natural killer (NK) cell and T‐cell function through multiple mechanisms and promote the expansion of regulatory T (Treg) cells, ultimately fostering an immunosuppressive tumor‐permissive microenvironment [33, 34, 35, 36]. These processes lead to functional exhaustion of immune effector cells, compromising the elimination of premalignant cells and facilitating immune evasion (Figure 1).

Defective immune surveillance is also well established as a central mechanism in the development of hematologic malignancies. T‐cell exhaustion, suppression of NK cell activity, and pathological expansion of myeloid‐derived suppressor cells have all been documented as contributors to the immunosuppressive milieu that enables malignant hematopoietic cells to escape immune control [37, 38].

In patients with vasculitis, downregulation of PD‐L1 promotes sustained overactivation, leading to exhaustion of effector T cells and diminishing the host's capacity to eradicate aberrant cells. At the same time, chronic inflammatory stimulation of the bone marrow hematopoietic niche increases the likelihood of genetic alterations in hematopoietic stem and progenitor cells. When such mutated cells acquire a proliferative advantage under compromised immune surveillance, they may ultimately evolve into hematologic malignancies (Figure 1).

#### 2.2.3 Genetic Susceptibility

Genetic susceptibility to BD is strongly linked to the human leukocyte antigen (HLA)‐B*51 allele [39, 40, 41]. Carriers of this allele are prone to sustaining a chronic pro‐inflammatory microenvironment, driven by excessive activation of Th1 and Th17 cells, as well as functional impairment of Treg cells [40, 42]. This inflammatory milieu promotes the activation of macrophages and neutrophils, leading to the overproduction of ROS and reactive nitrogen species [43, 44]. These reactive intermediates can directly induce oxidative DNA damage [43] and compromise the genomic stability of vascular endothelial cells [45]. Downregulation of HLA class I molecules, including HLA‐B*51, impairs antigen presentation, a central mechanism that facilitates tumor cells’ evasion of CD8^+^ T‐cell–mediated immune surveillance [46]. On this basis, HLA‐B*51–positive BD patients may be at increased risk of malignancy due to sustained inflammatory stress. At the same time, tumor cells may further exploit immune selective pressure by downregulating this allele to facilitate immune escape (Figure 1). Such mechanisms could contribute to resistance to immunotherapy and poorer clinical outcomes in patients with concomitant malignancies.

HLA‐B*52 is a well‐established susceptibility allele for TAK [47, 48, 49]. A multicenter cohort study of Japanese women identified HLA‐B*52 as an independent genetic risk factor for cervical cancer (odds ratio = 1.60, 95% CI 1.38–1.86), an effect attributed mainly to the p.Tyr171His variant located within the peptide‐binding groove of the HLA‐B molecule [50]. However, this association has not yet been confirmed in other ethnic populations or geographic settings. Evidence from studies on HLA‐B*52:01 in the context of human immunodeficiency virus infection suggests that this allele plays a role in viral immune regulation and may facilitate immune evasion [51, 52]. Therefore, it is plausible that the p.Tyr171His variant reduces the efficiency of human papillomavirus antigen presentation, impairing immune clearance of infected cells and increasing susceptibility to cervical cancer (Figure 1). However, this hypothesis remains to be tested by experiments.

HLA‐DRB1*04 has also been identified as a susceptibility allele for GCA [53, 54] (Figure 1). Although GCA predominantly affects older adults, this allele has been shown to act as a sex‐specific risk factor in female pediatric patients with acute lymphoblastic leukemia. It is associated with earlier disease onset [55]. In breast cancer, HLA‐DRB1*04 has been reported to influence HLA‐DR expression on tumor cells and to regulate the infiltration of tumor‐associated T cells, remodeling the tumor immune microenvironment and potentially promoting disease progression [56].

## 3 Clinical Features and Potential Mechanisms of Tumor‐Associated Vasculitis

### 3.1 Clinical Characteristics of Tumor‐Associated Vasculitis by Different Cancer Types

The Term “Tumor‐associated Vasculitis” Was Formally Introduced at the 2012 Chapel Hill Consensus Conference, Where It Was Defined as Vasculitic Involvement of Multiple Organ Systems, Directly or Indirectly Induced by an Underlying Malignancy [2]. In Line with this Concept, the 2022 Recommendations of the European Alliance of Associations for Rheumatology for the Management of AAV Advocate Expanded Malignancy Screening in Cases of Refractory Disease. These Guidelines Stress the Importance of Maintaining a High Index of Suspicion for Occult Tumors in Patients with Vasculitis Who Demonstrate Poor Therapeutic Response or atypical Clinical Features, Once Other Secondary Causes Have Been Excluded [57]. Guided by these Recommendations, the Following Inclusion Criteria Were Applied: (1) a Confirmed Diagnosis of Malignancy; (2) Clinical Manifestations of Vasculitis Fulfilling Established Diagnostic Criteria; (3) Significant Improvement of Vasculitic Symptoms Following Antitumor Therapy, Regardless of the Temporal Sequence of Diagnoses; and (4) Exclusion of Alternative Causes of Vasculitis. Using these Criteria, 22 Cases of Tumor‐associated Vasculitis Were Identified. Although the Cohort Size Is Limited, It Provides Meaningful Insights into the Clinical Presentation of this Entity (Table 2). Cases in Which Hematologic Malignancies or Lung Cancer Mimic Vasculitic Syndromes Have Been Reported More Frequently in the Literature; these Cases Are Summarized to Aid Clinical Recognition and Inform Diagnostic and Management Strategies (Table 2).

#### 3.1.1 Clinical Features of Vasculitis Associated with Hematological Malignancies

Analysis of Vasculitis Associated with Hematologic Malignancies Indicates That Cutaneous Involvement Is a Common Initial Presentation, Often Accompanied by Abnormalities in Hematologic Parameters. Case Reports by Broccoli and Moyers et al. describe Patients with Hairy Cell Leukemia Who Developed Palpable Purpura Predominantly Affecting the Lower Extremities; Histopathological Examination Confirmed Leukocytoclastic Vasculitis, and Laboratory Findings Revealed Pancytopenia [58, 59]. Similarly, Atallah and Kolodziejczyk et al. reported Cases of Chronic Myeloid Leukemia Presenting with Ulcerative Skin Lesions or Diffuse Subcutaneous Nodules, in Which Laboratory Assessments Demonstrated Leukocytosis or Thrombocytosis [60, 61].

The distribution of vasculitic involvement appears to vary according to the underlying hematologic malignancy. In particular, vasculitis associated with Hodgkin lymphoma shows a predilection for the central nervous system. Lopez‐Chiriboga et al. described a 25‐year‐old woman presenting with headache and mixed aphasia, in whom magnetic resonance imaging revealed white matter hyperintensities and subsequent brain biopsy confirmed granulomatous vasculitis [62]. A comprehensive review of 26 cases of Hodgkin lymphoma–associated vasculitis further supported this pattern, identifying headache and cognitive dysfunction as the most frequent presenting features [63].

Patterns of therapeutic response in these cases are especially informative. Multiple reports demonstrate that effective treatment of the underlying malignancy is often accompanied by resolution of vasculitic manifestations. For instance, cladribine therapy in patients with hairy cell leukemia achieved both hematologic remission and complete resolution of purpura [58, 59]. Similarly, subcutaneous nodules in chronic myeloid leukemia resolved entirely following treatment with imatinib [61]. In cases of central nervous system vasculitis associated with Hodgkin lymphoma, combination chemotherapy resulted in significant improvement in both clinical symptoms and radiologic findings [62]. These observations support a therapeutic strategy that simultaneously targets the malignancy and the associated inflammatory process.

#### 3.1.2 Clinical Features of Vasculitis Associated with Lung Cancer

Case reports of vasculitis associated with lung malignancies suggest a predominance in older patients, with palpable purpura of the lower extremities representing a frequent clinical manifestation (Table 2). A significant and potentially distinguishing laboratory feature in these cases is the dissociation between inflammatory markers. Patients often demonstrate significantly elevated erythrocyte sedimentation rate (ESR), typically 69 to 120 mm/h, while C‐reactive protein (CRP) levels remain within normal limits or are only mildly elevated. This pattern contrasts with primary vasculitis, in which ESR and CRP usually rise in parallel. For example, Scopelliti et al. described a 77‐year‐old patient with lung adenocarcinoma who had an ESR of 120 mm/h with a normal CRP level [64]. Similarly, Aguiar et al. reported a patient with a carcinoid tumor presenting with an ESR of 90 mm/h and a CRP level of 24 mg/dL [65]. In comparison, Pantoja Zarza et al. documented a case of squamous cell carcinoma with an ESR of 69 mm/h and a CRP level of 10.4 mg/dL [66].

From a therapeutic perspective, corticosteroid treatment may provide temporary symptomatic improvement; however, long‐term outcomes are determined mainly by effective management of the underlying malignancy. Aguiar et al. observed complete resolution of vasculitic manifestations following surgical resection of the carcinoid tumor [65]. However, patients with advanced‐stage lung cancer frequently experienced unfavorable prognoses due to tumor progression, despite initial alleviation of vasculitis‐related symptoms [64]. Given the limited number of reported cases, larger cohort studies are needed to validate these observations and further clarify the clinical characteristics of lung cancer–associated vasculitis.

#### 3.1.3 Clinical Features of Vasculitis Associated with Other Solid Tumors

Although reported less often, several other solid malignancies, including esophageal, prostate, and breast cancers, have also been implicated in the development of secondary vasculitis. Esophageal carcinoma, for instance, has been associated with large‐vessel vasculitis, presenting with systemic manifestations such as fever and arthralgia [67]. Isolated reports have described prostate cancer–related secondary GCA, characterized by persistent fever, night sweats, and bilateral hip pain [68]. Breast cancer has been reported to precipitate AAV, with clinical features including cutaneous eruptions and renal involvement [69]. In many of these cases, vasculitic manifestations improved substantially following effective antitumor treatment, lending support to the concept that tumor‐derived antigens may act as immunologic triggers for vasculitis (Figure 1).

### 3.2 Candidate Mechanisms Underlying Tumor Associated with Vasculitis

Multiple interconnected mechanisms likely underlie malignancies’ ability to mimic the clinical manifestations of vasculitis. These include immune complex deposition, pro‐inflammatory alterations within the tumor microenvironment, and direct tissue or vascular infiltration by neoplastic cells. Rather than operating in isolation, these processes frequently interact and reinforce one another during disease evolution, contributing to vasculitis‐like clinical presentations.

#### 3.2.1 Immune Complex Deposition as a Trigger

Tumor‐associated antigens may promote vascular inflammation by forming circulating immune complexes. In hairy cell leukemia and chronic myeloid leukemia, Broccoli and Kolodziejczyk et al. described leukocytoclastic vasculitis and PAN arising secondary to immune complex deposition [58, 61]. In lung adenocarcinoma, Scopelliti et al. reported the presence of anti‐endothelial cell antibodies, with vascular biopsy specimens demonstrating granulomatous inflammation. Surgical resection of the tumor normalized anti‐endothelial cell antibody titers and led to complete resolution of vasculitic manifestations, suggesting potential molecular mimicry between tumor antigens and endothelial components [65]. Although direct antigenic homology was not formally demonstrated, the close temporal relationship between antibody disappearance and clinical remission supports a combined pathogenic mechanism involving immune complex–mediated injury and molecular mimicry (Figure 1).

#### 3.2.2 Oxidative Stress and Immune Dysregulation in the Tumor Microenvironment

In addition to immune complex–mediated mechanisms, dysregulation of the tumor microenvironment may play a significant role in the development of vasculitic changes. As reviewed by Didion, IL‐6 can activate NADPH oxidase through the Janus kinase–signal transducer and activator of transcription 3 (JAK/STAT3) pathway, leading to increased superoxide anion generation and reduced nitric oxide (NO) bioavailability. These effects promote endothelial injury and upregulate intercellular adhesion molecule‐1 expression, facilitating the recruitment of inflammatory cells to the vascular wall [70].

In another illustrative case, Oka et al. described a tumor microenvironment characterized by a shift toward Th2 predominance, with elevated IL‐4 (50.3 pg/mL) and IL‐5 (56.1 pg/mL) levels, which synergized with IL‐6. This cytokine profile promoted eosinophil activation and immune complex deposition, exacerbating fibrinoid necrosis. Following treatment with bortezomib, IL‐6 levels declined to 3.4 pg/mL, accompanied by significant improvement in vasculitic manifestations, underscoring the central role of inflammatory cytokines in driving vasculitis‐like pathology [71] (Figure 1).

#### 3.2.3 Tumor Invasion into Vascular Structures

Direct invasion of vascular structures by malignant cells constitutes another plausible mechanism for the development of secondary vasculitis (Figure 1). Experimental work by Huang et al. demonstrated that integrin‐mediated adhesion of tumor cells to endothelial surfaces can activate focal adhesion complexes, disrupt intercellular junctions, and trigger pro‐inflammatory signaling pathways [72]. Complementary findings by Niland et al. identified matrix metalloproteinases, including significant matrix metalloproteinase‐14, as key mediators of basement membrane collagen degradation, exposing subendothelial components and promoting complement activation and immune cell recruitment [73]. Although clinical validation of these invasion‐related mechanisms remains limited, they provide a biologically plausible framework through which neoplastic infiltration may compromise vascular integrity, induce the release of damage‐associated molecular patterns, and provoke localized inflammatory responses culminating in secondary vasculitis.

## 4 Future Directions

This review provides a comprehensive synthesis of the clinical features and pathogenic mechanisms underlying both vasculitis induced by malignancy and malignancies arising in the context of vasculitis. To facilitate understanding, a schematic overview (Figure 1) has been constructed to illustrate the principal pathways involved and the shared contribution of genetic susceptibility factors, offering a practical reference for clinical application.

Future efforts should focus on expanding sample sizes and conducting multicenter cohort studies to strengthen the evidence base. The integration of biomarker profiling and molecular subtyping approaches will be critical for delineating specific association patterns between distinct vasculitis subtypes and tumor development, as well as between different malignancies and the induction of vasculitis. Such advances will support more accurate risk stratification and precision‐oriented screening strategies. Similarly, there is a need to establish standardized protocols for assessing comorbid conditions, develop individualized screening programs for high‐risk populations, and optimize therapeutic strategies that balance effective disease control with the prevention and management of complications. Enhanced multidisciplinary collaboration among rheumatology, oncology, and related specialties will further improve early diagnostic accuracy and reduce the likelihood of missed or delayed diagnoses. Greater emphasis on long‐term follow‐up, comprehensive management, and patient education, particularly regarding awareness of comorbidities and recognition of atypical symptoms, will facilitate earlier detection and timely intervention. These measures will contribute to the progressive refinement of diagnostic and therapeutic frameworks for vasculitis‐associated malignancies, ultimately improving patient quality of life and long‐term outcomes.

## Conflicts of Interest

The authors declare no conflicts of interest.

## Data Availability

The authors have nothing to report.
